# Nutritional and Pharmacological Strategies to Regulate Microglial Polarization in Cognitive Aging and Alzheimer’s Disease

**DOI:** 10.3389/fnagi.2017.00175

**Published:** 2017-06-07

**Authors:** Emiliano Peña-Altamira, Sabrina Petralla, Francesca Massenzio, Marco Virgili, Maria L. Bolognesi, Barbara Monti

**Affiliations:** Department of Pharmacy and Biotechnology, University of BolognaBologna, Italy

**Keywords:** immunomodulation, microglia, cognitive impairment, aging, Alzheimer’s disease, drug therapy, bioactive compounds, nutrition

## Abstract

The study of microglia, the immune cells of the brain, has experienced a renaissance after the discovery of microglia polarization. In fact, the concept that activated microglia can shift into the M1 pro-inflammatory or M2 neuroprotective phenotypes, depending on brain microenvironment, has completely changed the understanding of microglia in brain aging and neurodegenerative diseases. Microglia polarization is particularly important in aging since an increased inflammatory status of body compartments, including the brain, has been reported in elderly people. In addition, inflammatory markers, mainly derived from activated microglia, are widely present in neurodegenerative diseases. Microglial inflammatory dysfunction, also linked to microglial senescence, has been extensively demonstrated and associated with cognitive impairment in neuropathological conditions related to aging. In fact, microglia polarization is known to influence cognitive function and has therefore become a main player in neurodegenerative diseases leading to dementia. As the life span of human beings increases, so does the prevalence of cognitive dysfunction. Thus, therapeutic strategies aimed to modify microglia polarization are currently being developed. Pharmacological approaches able to shift microglia from M1 pro-inflammatory to M2 neuroprotective phenotype are actually being studied, by acting on many different molecular targets, such as glycogen synthase kinase-3 (GSK3) β, AMP-activated protein kinase (AMPK), histone deacetylases (HDACs), etc. Furthermore, nutritional approaches can also modify microglia polarization and, consequently, impact cognitive function. Several bioactive compounds normally present in foods, such as polyphenols, can have anti-inflammatory effects on microglia. Both pharmacological and nutritional approaches seem to be promising, but still need further development. Here we review recent data on these approaches and propose that their combination could have a synergistic effect to counteract cognitive aging impairment and Alzheimer’s disease (AD) through immunomodulation of microglia polarization, i.e., by driving the shift of activated microglia from the pro-inflammatory M1 to the neuroprotective M2 phenotype.

## Microglia and Their Polarization

Microglia are the resident mononuclear phagocytes of the central nervous system and constitute about 5%–20% of total brain cells displaying regional differences in density (Lawson et al., 1990). These cells are known for their plastic capability and the functional characteristic depending on their activation state; indeed, it is not difficult to find morphological changes these cells undergo depending on their function (Szabo and Gulya, 2013). Under physiological conditions, microglial cells are in a “resting” state of non-activation, and cell morphology is characterized by numerous processes that originate from the soma with distal arborization (Hailer et al., 1996). On the other hand, under conditions of stress, inflammation, injury or upon the effect of certain signals, microglia change their morphology and activation state, from a non-activated to an activated state (Stence et al., 2001). Microglial cells enlarge their cell body and their ramifications become shorter and less arborized. This morphological mutation can induce phagocytic functions and amoeboid ability that allows cells to move toward sites of injury (Tzeng and Wu, 1999). A typical feature of this state is the activation of microglia and subsequent release of inflammatory mediators that lead to an increased oxidative and nitrosative stress. This condition promotes the inflammatory process, and persisting microglial activation in this condition shows to be harmful to the nervous tissue (Csuka et al., 2000; Polazzi and Monti, 2010). Activated microglia have two different phenotypes: M1 and M2. Classically activated M1 microglia, activated by LPS or IFN-γ, have pro-inflammatory, neurotoxic properties, inhibiting the proliferation of lymphocytes; M1 activated macrophages secrete proinflammatory cytokines, such as interleukin IL-1α, IL-1β, tumor necrosis factors (TNF) and nitric oxide (NO). Alternatively activated M2 microglia are able to repair small damage, have an anti-inflammatory phenotype, contributing to trophic support of neurons, possess the ability to degrade toxic aggregates and increase the neuroprotective functions thanks to anti-inflammatory interleukin production (Michelucci et al., 2009; Choi et al., 2017). The M2 phenotype is further divided in three subtypes; M2a, M2b, M2c. They may have comparable biochemical functions, but are different in the mechanisms of action. The shift to the M2a subtype is driven by IL-3 and IL-4 and is involved in collagen formation, tissue repair and immunity against parasites whereas the transition to the M2b subtype is driven by the activation of Toll-like receptor (TLRs) agonists and is able to recruit regulatory T-cells. On the other hand, the M2c phenotype, induced by IL-10, TGFβ1 and glucocorticoids, is involved in the repair of damage and injury (Chhor et al., 2013; Mecha et al., 2015).

Regarding the beneficial or detrimental role of activated microglia on neuronal survival, the most accredited hypothesis today is that microglia may have a dual role, both neuroprotective and neurotoxic, depending on their activation state, which in turn depends on the nature, length and extension of the insult (Ransohoff and Perry, 2009). *In vitro* studies in cell cultures have shown the ambivalent role of microglial cells on neurons; neuroprotective, but also neurotoxic, while *in vivo* studies mainly support the neuroprotective potential of activated microglia (Streit, 2002).

## Cognitive Deficits in Aging and AD

Aging is defined as “the gradual change in an organism that leads to increased risk of weakness, disease and death” (Merriam-Webster thesaurus). It takes place all throughout an organism and the brain is no exception. Aging leads to reduced brain size, neurotransmitter receptor alterations, dendrite loss/regression and electrophysiological changes such as cortical spreading depression alterations, possibly connected also to cortical microglial reactivity, as shown by Iba-1 immunolabeling (Landfield et al., 1978; Earnest et al., 1979; Jacobs et al., 1997; Hof et al., 2002; Duan et al., 2003; Luebke et al., 2004; Batista-de-Oliveira et al., 2012; Lima et al., 2014). These alterations lead to what is normally called “age related cognitive decline”. Human cognitive function can be classified in basic cognitive functions: attention, working memory, long-term memory, perception; and higher-level cognitive functions: speech and language, decision making, executive control (Glisky, 2007). However, much research on cognitive function has mainly focused on memory, and this could account for variability between aged individuals. The term “mild cognitive impairment” (MCI) was first introduced with the Global Deterioration Scale (Reisberg et al., 1982) for those individuals whose cognitive performance is below normal according to age-matched healthy individuals, especially regarding memory-based performance. The term was further refined in 2004 by the International Working Group on MCI (Winblad et al., 2004), in which affected individuals are considered those that show evidence of cognitive decline after appropriate testing, but maintain normal everyday life activities and functions. MCI appears to be a risk factor for developing dementia as shown by a Chinese study in which about 30% of patients with MCI developed dementia within 2 years and high plasma C-reactive protein levels were associated with accelerated cognitive decline and increased risk of dementia (Xu et al., 2009). Moreover, in another recent study, about 20% of patients with MCI developed Alzheimer’s disease (AD) within 2 years after diagnosis, as assessed through biochemical and magnetic resonance imaging (MRI) performed for brain volumetric assessment, among which hippocampal volume (Nesteruk et al., 2016). AD is a neurodegenerative disease characterized by progressive cognitive decline, present both as familial and sporadic cases. Aβ production and processing alterations are thought to be one of the causes that trigger the disease. Post-mortem brain studies have shown that AD pathology hallmarks are the deposition of extracellular Aβ plaques as well as intracellular neurofibrillary tangles (Lantos et al., 1992). Familial AD which accounts for 2% of all cases and may have a disease onset as early as 40–50 years, is caused by mutations in the amyloid precursor protein (APP) gene and presenilin 1–2 genes prevalently (Karlinsky et al., 1992; Levy-Lahad et al., 1995; Sherrington et al., 1995). However, also rare TREM2 receptor mutations increase the risk of developing AD (Guerreiro et al., 2013; Jonsson et al., 2013). TREM2 is an innate immune receptor expressed by macrophages and dendritic cells, among other cell types, while in the central nervous system it is expressed mainly by microglia (Hickman and El Khoury, 2014) and is involved in inflammation and phagocytosis. Mutations in TREM2 may impair phagocytosis (Kleinberger et al., 2014), supporting microglial involvement in AD pathology. On the other hand, sporadic AD shows late disease onset around 60–70 years for which the apoliprotein E type 4 (APOE-ε-4) allele has been identified as a major risk factor (Corder et al., 1993). Currently, there is no cure available for AD, yet AD is responsible for 60%–80% of all dementia cases (Alzheimer’s international statistics). Nearly 46.8 million people worldwide were affected by dementia in 2015 and this number is expected to reach 131.5 million cases by 2050 (Alzheimer’s international statistics). Thus, dementia represents a burden to society and to healthcare systems.

## Immunomodulation as A Promising Therapeutic Strategy to Counteract Cognitive Impairment

**Figure 1:**
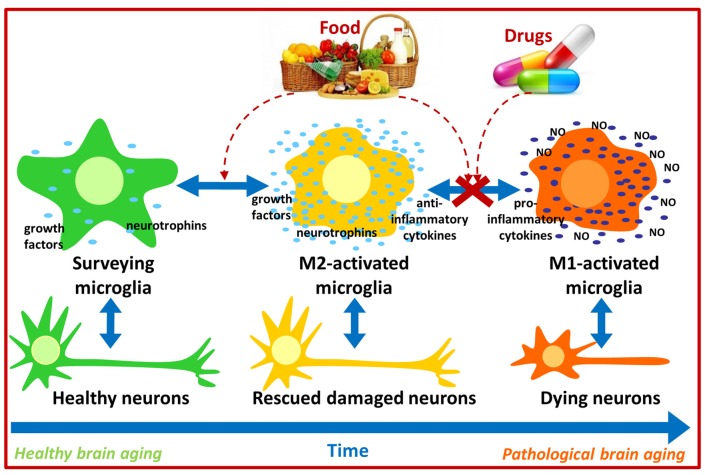
Possible pharmacological and nutritional approaches towards immunomodulation to counteract pathological brain aging. Food bioactive compounds and/or synthetic compounds may influence microglial activation state contributing to neuronal survival and thus improve cognitive function during aging.

Under physiological conditions, immune responses exert positive effects on the brain by regulating neuroplasticity, learning and memory, while injury or chronic stress lead to the increased production of inflammatory molecules such as IL-1, IL-6 and TNF-α which may disrupt neurotrophic factor production/signaling and impair learning and memory (Schneider et al., 1998; Parish et al., 2002; Avital et al., 2003; Balschun et al., 2004; Golan et al., 2004; Soiampornkul et al., 2008). Interestingly, increased pro-inflammatory IL-6 and reduced anti-inflammatory IL-10 levels have been detected in brains from aged mice (Ye and Johnson, 1999, 2001). Moreover, immune status can also influence brain function in humans as the Hoorn Study evidenced that increased levels of inflammatory plasma markers (TNF-α, IL-6, IL-8, C-reactive protein) were associated with cognitive decline (Heringa et al., 2014). Because M1 activated microglia produce inflammatory cytokines, and these seem to induce cognitive impairment, immunomodulation strategies aiming to attenuate M1 microglial activation or induce an M1 to M2 microglial shift may contribute to counteract, at least partially, cognitive impairment in aging and in neurodegenerative diseases such as AD. Thus pharmacological and nutritional approaches targeting immunomodulation are currently being developed, and a remarkable amount of data has been produced recently (Figure 1).

## Pharmacological Approaches Towards Immunomodulation and Their Potential Application in Aging/AD

Therapeutic strategies aimed to modify microglia polarization, and therefore leading to immunomodulation, are currently being developed. Pharmacological approaches able to shift microglia from the M1 pro-inflammatory to the M2 neuroprotective phenotype could become new therapeutic tools in neurodegenerative diseases such as AD and also in normal brain aging. In the following sections we discuss on potentially interesting and successful immunomodulatory targets including glycogen synthase kinase-3 (GSK3) β, b-site APP-cleaving enzymes (BACEs), Janus kinase (JNK), phosphodiesterases (PDEs), AMP-activated protein kinase (AMPK), histone deacetylases (HDACs) and peroxisome proliferator-activated receptor gamma (PPAR-γ) according to currently available evidence.

### GSK-3 β

GSK-3 β is a serine/threonine kinase, involved in several signaling pathways such as cell proliferation and inflammation (Cui et al., 1998; Park et al., 2006). Notably, GSK-3β appears to be responsible for tau hyperphosphorylation (Sperber et al., 1995), detaching tau from microtubules and inducing tau precipitation as intracellular neurofibrillary tangles (NFTs; Ferrer et al., 2002), a hallmark of AD. Moreover, GSK-3β has been shown to mediate the release of inflammatory cytokines in LPS-activated microglia *in vitro* (Green and Nolan, 2012), thus GSK-3 could be a promising target. Tideglusib (NP031112), a thiadiazolidinine derivative, prevents inflammation and neurodegeneration in a kainic-acid inflammation rat model (Luna-Medina et al., 2007) and has reached phase II clinical trials on AD patients (Clincaltrials.gov identifier NCT00948259), appearing safe though not effective enough (del Ser et al., 2013; Lovestone et al., 2015). In another study, two triazolopyridine derivative GSK-3 inhibitors (C-7a and C-7b) reduced Aβ neurotoxicity and tau hyperphosphorylation *in vitro* and improved cognitive deficits in a transgenic AD mouse model (Noh et al., 2013), though no anti-inflammatory effects were investigated. More recently, triazinones displayed i*n vitro* anti-GSK-3 and anti-BACE-1 activity plus neuroprotective and neurogenic effects besides good brain permeability *in vivo* (Prati et al., 2015), however no anti-inflammatory data are currently available. Lastly, L807mts, a highly selective GSK-3 peptide derivative inhibitor, reduced Aβ levels, reduced inflammation and enhanced autophagy in a transgenic AD mouse model (Licht-Murava et al., 2016).

### BACEs

Aβ aggregates are generated by the cleavage of the membrane-associated APP by α, β and γ-secretases. Through two successive proteolytic cleavages by α-secretase and γ-secretase, a harmless peptide, p3, is produced. On the other hand, β-secretase operates a different cleavage leading to the production of two neurotoxic peptides of 40 and 42 amino acids each, called Aβ40 and Aβ42, respectively. The first β–secretase identified was BACE-1, a type I transmembrane aspartic protease (Vassar et al., 1999). Because Aβ can induce neuroinflammation, BACE-1 inhibitors can contribute indirectly to inhibiting microglial M1 activation and research on their potential application is ongoing. *In vivo* treatment with the aminoisoindole AZ-4217, a BACE-1 inhibitor, resulted in long term reduced Aβ deposition in an APP transgenic cerebral amyloidosis mouse model (Eketjäll et al., 2013). In another study, treatment with the BACE inhibitor RO5508887 reduced amyloid plaque load and formation in an AD transgenic mouse model and combined treatment with an anti-Aβ antibody (gantenerumab) further enhanced these effects (Jacobsen et al., 2014), however the impact on cognition was not investigated. Also, as previously stated, triazinones displayed anti-GSK-3 and anti-BACE-1 activity (Prati et al., 2015) while NB-360, another BACE-1 inhibitor was able to block Aβ deposition and the accumulation of inflammatory cells in a transgenic AD mouse model (Neumann et al., 2015). Noteworthy, a clinical trial to study the effect of the BACE-1 inhibitor LY3202626 on patients with mild AD dementia, as of February 2017 was recruiting patients (Clinicaltrials.gov identifier NCT02791191).

### JNK

Mitogen-activated protein kinases (MAPKs) are serine–threonine kinases that mediate intracellular signaling. JNK is a major cellular stress response protein induced by oxidative stress and its activation is believed to be an early event in AD (Zhu et al., 2001). Among the MAPKs, JNK is one of the essential mediators of microglial proinflammatory functions (Waetzig et al., 2005), it is a component of signaling pathways that lead to inflammation, and it can control the synthesis and release of proinflammatory molecules by LPS-activated microglia (Hidding et al., 2002). Moreover, the JNK-AP1 signaling pathway mediates the increased expression of inflammatory genes induced by Aβ peptides in human brain endothelial cells and in AD brain (Vukic et al., 2009). Thus JNK is an attractive target for AD prevention/therapy. In one study, treatment of LPS-activated primary rat microglia with conserved dopamine neurotrophic factor (CDNF) resulted in reduced production of inflammatory cytokines by suppressing the phosphorylation of JNK and thus inhibiting JNK signaling (Zhao et al., 2014). Therefore, CDNF use *in vivo* seems promising. Another recent *in vivo* study showed that administration of the JNK inhibitor SP600125 resulted in cognitive deficit improvement in a transgenic AD mouse model by reducing Aβ production, inflammatory responses and synaptic loss (Zhou et al., 2015). Interestingly, *in vivo* administration of the small molecule LX2343 improved cognitive deficits in a transgenic AD mouse model by inhibiting both JNK and BACE-1 activity, reducing Aβ production and promoting autophagy (Guo et al., 2016), though no direct anti-inflammatory mechanisms were investigated.

### PDEs

PDEs act as regulators of intracellular signaling cascades through the control of 2 second messengers, cyclic adenosine monophosphate and cyclic guanosine monophosphate. Recent findings point to PDE inhibitors as compounds able to affect different mechanisms underlying AD, as shown by active ongoing research with these molecules. Administration of sildenafil, a PDE-5 inhibitor, improved cognitive deficits, reduced Aβ levels and neuroinflammation in a transgenic AD mouse model (Zhang et al., 2013). Moreover, administration of another PDE-5 inhibitor, yonkenafil, resulted in improved cognitive function, increased neurogenesis in the dentate gyrus and reduced activation of microglia and astrocytes in a transgenic AD mouse model (Zhu et al., 2015), therefore showing also anti-inflammatory effects. In another study, the PDE-7 inhibitor S14 improved cognitive impairment, reduced Aβ deposition and tau phosphorylation in a transgenic AD mouse model (Perez-Gonzalez et al., 2013). Also in an *in vitro* study, the PDE-3 inhibitor cilostazol reduced Aβ production by increasing ADAM10 expression (Lee et al., 2014), though no anti-inflammatory effects were investigated. Noteworthy, a phase IV clinical trial to examine the additive effects of cilostazol on donepezil-treated mild to moderate AD patients has been completed (Clinicaltrials.gov identifier NCT01409564) and in a very recent small clinical trial, cilostazol administration as an add-on therapy reduced cognitive decline in AD patients (Tai et al., 2017). In another study, administration of GEBR-7b, a PDE-4D inhibitor, improved cognitive deficits in a transgenic AD mouse model (Sierksma et al., 2014) though the underlying mechanisms of action remain to be discovered. Lastly, in a very recent *in vivo* study, administration of the PDE-4 inhibitor FFM reversed cognitive deficits in a transgenic AD mouse model by increasing CREB phosphorylation and BDNF levels and reducing inducible NO synthase (iNOS), TNF-α and IL-1β levels (Guo et al., 2017).

### AMPK

AMPK is a highly conserved energy sensor involved in mitochondrial biogenesis, cellular stress responses and regulation of inflammation (Corton et al., 1994; Zong et al., 2002; Giri et al., 2004). Because overactivated AMPK has been reported to accumulate in neurons containing tangles in AD brains (Vingtdeux et al., 2011) and AMPK can phosphorylate tau protein (Domise et al., 2016), AMPK may represent an interesting target, though its role in AD is not fully understood. While inhibition of AMPK with the compound C (CC) improved hippocampal synaptic plasticity impairment induced by Aβ in a transgenic AD mouse model (Ma et al., 2014) AMPK activators have also shown beneficial effects in AD models. An example are the anti-epileptic drugs topiramate and levetiracetam which improved cognitive deficits, reduced Aβ production, increased the activation of AMPK and inhibited HDAC activity in a transgenic AD mouse model (Shi et al., 2013). Moreover, a phase II levetiracetam clinical trial on MCI patients was completed in 2012, though no results were posted (Clinicaltrials.gov identifier NCT01044758). Also, while not tested in AD, telmisartan, an angiotensin II type 1 receptor blocker, increased brain AMPK activation and microglial M2 gene expression in an LPS-induced neuroinflammation mouse model (Xu et al., 2015) thus its application in AD may deserve attention. In another study, administration of the AMPK activator AICAR, improved cognitive deficits in a streptozotocin-induced AD rat model (Du et al., 2015) by restoring mitochondrial functions, though no anti-inflammatory effects were investigated. Lastly, treatment with the small molecule THSG exerted anti-inflammatory effects on LPS-activated microglia by reducing iNOS, COX-2, TNF-α and IL-6 levels and increased AMPK phosphorylation levels, thus confirming AMPK activation (Park et al., 2016).

### HDACs

The effects of histone acetylation on the activation of gene expression were unknown until the 1960s (Allfrey et al., 1964). Acetylation reduces the positive charge of basic histone proteins thus decreasing their interaction with DNA and allowing gene expression. More in detail, this process is finely regulated by several Histone Acetyl Transferases (HATs) and HDACs. Recently, the effects of HDAC inhibitors on immunomodulation have increasingly generated interest due to a potential role in immunotherapy. In fact, class I HDAC inhibitors have been reported to increase transcription of neuronal genes, provide neuroprotective effects, and enhance cognitive abilities. Moreover, class I HDAC inhibitors such as valproic acid (VPA), trichostatin A (TSA), suberoylanilide hydroxamic acid (SAHA), sodium phenylbutyrate (SB) and MS-275 have been shown to enhance neurite outgrowth, synaptic plasticity, neurogenesis, neuronal differentiation and axon regeneration in cultured neurons and *in vivo* (Laeng et al., 2004; Siebzehnrubl et al., 2007; Koriyama et al., 2014). In one *in vivo* study, administration of the HDAC inhibitor SB, prevented cognitive deficits and reduced Aβ and GFAP levels, suggesting an anti-inflammatory effect in a transgenic AD mouse model (Ricobaraza et al., 2011). Another *in vivo* study showed that treatment with the benzamide HDAC inhibitor MS-275 improved cognitive deficits and reduced microglial activation and Aβ accumulation in a transgenic AD mouse model (Zhang and Schluesener, 2013). Lastly, in another *in* *vivo* study, SAHA treatment restored H3 acetylation and BDNF levels (Sharma and Taliyan, 2016) in mice fed a fat-enriched diet which developed insulin resistance-induced cognitive decline. While not being an AD study, insulin resistance is considered a risk factor for AD (Fava et al., 2017) and thus SAHA may deserve attention in AD research.

### PPAR-γ

PPAR-γ is a nuclear receptor that, after binding peroxisome proliferators, binds to specific DNA PPAR response elements regulating the expression of genes involved in fatty acid peroxisomal beta-oxidation (Dreyer et al., 1993). Current evidence shows that PPAR-γ activation may prove useful in AD therapy. In one *in vitro* study pioglitazone, a PPAR-γ agonist, inhibited iNOS and the production of IL-1β, TNF-α and IL-6 in LPS-activated HAPI microglia (Ji et al., 2010). Unfortunately, a phase II pioglitazone clinical trial performed in mild to moderate AD patients completed in 2009 produced no results (Clinicaltrials.gov identifier NCT00982202). In another *in vitro* study, the small molecule SNU-BP inhibited inflammatory cytokine production and iNOS in LPS-stimulated microglia by activating PPAR-γ. SNU-BP also increased IL-4 and arginase-1 expression, considered as M2 microglial phenotype markers (Song et al., 2016), thus SNU-BP may deserve *in vivo* testing as well. Interestingly, PPAR-γ agonists able to act on other targets are also being studied in AD therapy. Such is the case for T3D-959, a PPAR-γ and PPAR-δ agonist that reversed neurodegeneration in a streptozotocin-induced AD mouse model though no anti-inflammatory mechanisms were investigated (Tong et al., 2016). Noteworthy, a phase II clinical trial focused on TD3D-959 in mild to moderate AD patients as of June 2016 was being performed (Clinicaltrials.gov identifier NCT02560753). Lastly, MH84 a PPAR-γ agonist/γ-secretase modulator, reduced Aβ42 in HEK293APPwt cells, characterized by elevated Aβ42 levels, and improved mitochondrial dysfunction (Pohland et al., 2016), thus performing *in vivo* MH84 studies applied to AD therapy may be worth.

## Nutritional Approaches Towards Immunomodulation and Their Potential Application in Aging/AD

Interest in the role of diet in health developed after several epidemiological studies found an association between the Mediterranean diet and a lower risk of developing cardiovascular diseases and death (Menotti et al., 1996; Knoops et al., 2004). Nutritional approaches to target immunomodulation could become interesting strategies given that foods we consume on a daily basis, especially plant-based foods (widely present in the Mediterranean diet), besides providing nutrients contain also non-essential molecules, termed bioactive food compounds, able to exert multiple effects throughout the body by acting on different targets. Science has started understanding the effect of these molecules, and they could join the list of key players in immunomodulation strategies.

Bioactive compounds comprise a heterogeneous group of thousands of molecules. They can be classified into four/five major groups depending on authors: carotenoids (including carotenes and xantohpylls); phytosterols (including sterols and stanols); phenolic compounds (including chlorogenic acid); polyphenols (including flavonoids, stilbenes, lignans and curcuminoids) and sulfur compounds (including glucosinolates; Lozano et al., 2016).

Being a heterogeneous group of molecules with distinct chemical structures, bioactive compounds exert therefore different effects such as anti-inflammatory, antioxidant, hypoglycemic, cholesterol-lowering, estrogen mimicking, immunostimulant, neurogenic and neuroprotective effects.

Nutritional strategies based on the use of bioactive compounds can include dietary enrichment, by increasing the intake of foods containing naturally high amounts of bioactive compounds; intake of bioactive compound-enriched supplements, such as concentrated extracts or purified single molecules; intake of foods fortified with bioactive compounds. On the other hand, dietary supplements containing high amounts of bioactive compounds are already commercially available.

Given their multifaceted properties, many studies have focused on the effect of bioactive compounds which we summarize in the following sections and discuss on their potential use in aging/AD.

### Carotenoids

Well-known carotenoids such as β-carotene, lycopene and lutein can be found in carrots, tomatoes, peaches and peppers among other foods (Lozano et al., 2016). Lycopene, highly concentrated in tomatoes, was able to reduce BV-2 microglial cell activation after LPS treatment *in vitro* by reducing COX-2 expression (Lin et al., 2014). Moreover, lycopene exerted anti-inflammatory effects and enhanced cognitive performance in a Aβ42-induced AD rat model by downregulating inflammatory cytokines such as TNF-α and IL1-B (Sachdeva and Chopra, 2015). Also lutein, highly present in parsley, was able to reduce LPS-induced neuroinflammation in BV-2 microglial cells, by significantly inhibiting iNOS and cyclooxygenase-2 activity, as well as by reducing TNF-α and IL-1β production (Wu et al., 2015). However, no *in vivo* data on the anti-inflammatory effect of lutein are currently available. Interestingly, a human study involving nearly 7000 participants aged 50 or older found an association between high levels of serum lycopene and lutein and a lower risk of mortality by AD (Min and Min, 2014). Current research on AD and bioactive compounds has also focused on less-known carotenoinds. For example, fucoxanthin, a carotenoid abundant in brown seaweeds, exerted anti-inflammatory effects and reduced reactive oxygen species levels in Aβ42 treated BV2 microglial cells (Pangestuti et al., 2013). Moreover, in another *in vitro* inflammation-induced microglial cell culture study, fucoxanthin was able to inhibit the secretion of inflammatory cytokines after LPS treatment (Zhao et al., 2017). However, no *in vivo* data on the anti-inflammatory effect of fucoxanthin are currently available.

On the other hand, carotenoids contained in saffron, such as crocin and crocetin, exerted *in vitro* anti-inflammatory effects on LPS-treated rat brain microglial cells by inhibiting NO synthesis from iNOS and the production of inflammatory cytokines (Nam et al., 2010). Also, in another study, trans-crocetin was able to improve Aβ degradation in monocytes derived from AD patients (Tiribuzi et al., 2017) thus it would be interesting to determine whether trans-crocetin may induce the same effect in microglia. Remarkably, crocin intraperitoneal administration showed neuroprotective effects *in vivo*, by blocking Aβ-induced apoptosis in an AD animal model (Asadi et al., 2015) which means crocin is able to cross the blood brain barrier, despite its hydrophilic nature.

### Phytosterols

Phytosterols such as β-sitosterol, stigmasterol and campesterol can be found in vegetable oils, such as olive oil, cereals, legumes and nuts (Lozano et al., 2016). While a direct anti-inflammatory activity in AD has not been reported for most phytosterols, their anti-inflammatory effects may not have been thoroughly investigated. Stigmasterol, one of the most prevalent phytosterols in foods, exhibited beneficial effects *in vivo* in an AD animal model, mainly by reducing Aβ generation (Burg et al., 2013). In another *in vivo* study, a plant sterol-enriched diet was able to prevent cognitive impairment in SAMP8 mice, a non-transgenic AD animal model. This beneficial effect could derive from the fact that plant sterols may substitute lost cholesterol in SAMP8 mice brains (Pérez-Cañamás et al., 2016). AD studies have also focused on less common phytosterols. For example, spinasterol, isolated from *Aster scaber*, widely used in Korean cuisine, exerted anti-inflammatory effects on LPS-activated BV-2 microglial cells by upregulating heme-oxygenase-1 and reducing the production of TNF-α, IL-1β and PGE2 (Jeong et al., 2010). However, no anti-inflammatory effects for spinasterol in aging/AD models have been reported. Moreover, fucosterol, a phytosterol contained in brown seaweed, exerted anti-inflammatory effects on RAW264.7 macrophages by inhibiting the production of NO and the expression of iNOS and cyclooxygenase-2 (Jung et al., 2013). This suggests that fucosterol may exert the same anti-inflammatory effects on microglial cells. Interestingly, a recent *in vitro* study additionally shows that fucosterol may also exert BACE-1 inhibitory effects (Jung et al., 2016).

### Phenolic Compounds/Polyphenols

Phenolic compounds such as tyrosol, capsaicin, coumaric, caffeic and chlorogenic acid can be found in citrus, olives, oats and soybeans wheras polyphenols, the most heterogeneous group of bioactive compounds, which includes quercetin, rutin, flavan-3-ols, catechins, anthocyanins, flavones (luteolin), isoflavones (phytoestrogens such as genistein, daidzein), curcumin and lignans can be found in onions, blueberries, red wine and tea (Lozano et al., 2016). Chemically speaking, phenolic acids belong to the supercategory of polyphenols, and thus we describe several examples together. In one *in vitro* study, caffeic acid, naturally present in honeybee propolis, exerted anti-inflammatory effects on BV-2 microglia by inhibiting the expression of iNOS and cyclooxygenase-2 (Tsai et al., 2015). Moreover, caffeic acid oral administration improved cognitive deficits in an Aβ25–35-induced AD animal model, possibly by inhibiting lipid peroxidation and NO production (Kim et al., 2015).

In another study, artoindonesianin O, a phenolic compound found in mulberries, blocked Aβ-42 toxicity in an *in vitro* model of AD (Qiao et al., 2015). While no *in vivo* anti-inflammatory properties for artoindonesianin O have been reported, a recent report showed that artoindonesianin O is a potent lipooxygenase inhibitor, thus it could also play an anti-inflammatory role *in vivo* (Lang et al., 2016).

On the other hand, carnosic acid found in rosemary and sage, was able to inhibit LPS-induced activation of MG6 microglial cells *in vitro*, by reducing iNOS levels (Yanagitai et al., 2012). Moreover, carnosic acid was able to improve cognitive deficits in an Aβ40-induced AD rat model (Rasoolijazi et al., 2013). In another study, oral administration of ferulic acid, found in seeds and cereals, particularly flaxseed (Beejmohun et al., 2007), inhibited microglial activation in an Aβ42-induced AD mouse model by decreasing IFN-gamma levels in the hippocampus (Kim et al., 2004). Moreover, ferulic acid administration was able to improve cognitive deficits in a transgenic AD mouse model (Mori et al., 2013).

Interest in the application of other polyphenols normally present in foods for AD research has constantly increased. In fact, oral administration of oleuropein aglycone, found in olive leaves, significantly attenuated astrocyte and microglial activation in an Aβ42-induced AD rat model (Luccarini et al., 2014). Moreover, oleuropein aglycone oral administration also improved cognitive deficits and reduced Aβ42 plaque area and number in a transgenic AD mouse model (Pantano et al., 2017). Also resveratrol, a polyphenol found in red grapes and wine, was able to inhibit Aβ-induced activation of BV-2 microglial cells by reducing the production of inflammatory factors such as TNF-α, IL-1β and NO (Yao et al., 2015). Moreover, resveratrol administration showed anti-inflammatory and anti-apoptotic effects *in vivo* in an Aβ42-induced AD mouse model by inhibiting PDE-4 signaling (Wang et al., 2016). Interestingly, dietary resveratrol also extended life span of SAMP8 mice, a non-transgenic AD mouse model through sirtuin activation (Porquet et al., 2013). However, a resveratrol phase II clinical trial on patients with mild to moderate AD, resulted in no benefit to patients (Turner et al., 2015; Clinicaltrials.gov identifier NCT01504854).

In another study, curcumin, mainly found in turmeric, inhibited Aβ induced microglial activation *in vitro* (Shi et al., 2015). Moreover, curcumin administration improved cognitive deficits in an AD transgenic rat model, possibly through an anti-inflammatory effect by activating PPAR-gamma (Liu et al., 2016) and a phase II curcumin clinical trial on mild to moderate AD patients was concluded in 2009 though no results were posted (Clinicaltrials.gov identifier NCT00099710).

Regarding phytoestrogens, genistein, the main isoflavone found in soy, exerted anti-inflammatory effects on LPS-activated BV-2 microglia by blocking TLR4 signaling (Jeong et al., 2014). Moreover, genistein improved cognitive deficits in an AD mouse model (Bonet-Costa et al., 2016). However, oral administration of soy isoflavones (100 mg/day) to AD patients for 6 months resulted in no cognitive deficit improvement (Gleason et al., 2015; Clinicaltrials.org identifier NCT00205179). This could be due to the fact that genistein is metabolized by the gut microbiome to its most active metabolite, equol, only in presence of specific bacteria in the gut microbiome such as Slackia isoflavoniconvertens (Matthies et al., 2009).

In another study, anthocyanin *in vitro* treatment inhibited LPS-induced BV-2 microglial activation by reducing IL-1β levels (Meireles et al., 2016). Moreover, a diet supplemented with 1% anthocyanin extracts was able to prevent cognitive deficits in a transgenic AD mouse model (Yamakawa et al., 2016) whereas pomegranate polyphenol administration exerted anti-inflammatory effects on a transgenic AD mouse model by reducing TNF-α brain levels and microgliosis (Rojanathammanee et al., 2013).

Even the polyphenol epigallocatechin from green tea, attenuated Aβ EOC 13.31 microglial activation *in vitro* (Cheng-Chung Wei et al., 2016). While no *in vivo* anti-inflammatory effects for green tea catechins in AD have been reported, green tea epicatechin administration combined with treadmill exercise improved cognitive deficits in an AD transgenic mouse model (Zhang et al., 2016). Also, a phase II epigallocatechin clinical trial on early stage AD patients was completed on 2016 though no results have been published yet (Clinicaltrials.gov identifier NCT00951834).

Lastly, rutin, a citrus flavonoid, exerted anti-inflammatory and antioxidant effects in an AD transgenic mouse model, improving spatial memory (Xu et al., 2014).

Interestingly, one *in vivo* study showed that dietary polyphenols may exert in part their beneficial effects after being converted into phenolic acids by intestinal microbiota (Wang et al., 2015).

### Sulfur Compounds

Sulfur compounds such as glucosinolates (isothiocyanate, sulphoraphane) and Allium genus compounds (allin, allicin and ajoene) can be found in cabbage, broccoli, onions and garlic (Lozano et al., 2016). Sulforaphane, an isothiocyanate derived from glucoraphanin hydrolysis in broccoli, exerted anti-inflammatory effects *in vitro* on LPS-activated microglia by decreasing IL-1β, IL-6 and TNF-α expression (Brandenburg et al., 2010). Moreover, sulforaphane was able to extert anti-inflammatory effects against Aβ42-induced microglial activation in THP-1 macrophages through STAT-1 dephosphorylation and the activation of heme-oxygenase 1 (An et al., 2016). While no *in vivo* anti-inflammatory effects in AD disease have been reported for sulforaphane, sulforaphane administration exerted neuroprotective effects in an AD aluminum-induced mouse model (Zhang et al., 2015). Moreover, a very recent study showed that treatment with moringin, an isothiocyanate derived from the edible plant *Moringa oleifera*, exerted potent *in vitro* ant-inflammatory effects on LPS-activated RAW 264.7 macrophages (Giacoppo et al., 2017). Also, moringin showed *in vivo* anti-inflammatory effects in a Parkinson’s disease mouse model (Giacoppo et al., 2017) and thus it would be worth testing moringin also on aging and AD models.

## *In Vitro* and *In Vivo* Considerations

Microglial cells are able to exert neuroprotective effects towards neurons challenged with toxic insults. Evidence shows that this mechanism can be exerted either through direct contact or through factors secreted by microglia, as shown by *in vitro* studies (Polazzi et al., 2001, 2009, 2013). On the other hand, nanomolar concentrations of Aβ induced neuronal death in mixed neuron-glial cerebellum cultures, possibly through a microglia mediated mechanism (Neniskyte et al., 2011), thus outlining a multifaceted role of microglial activation. Of remarkable importance are the limitations of the *in vitro* and *in vivo* studies here discussed. Regarding both pharmacological and nutritional *in vitro* studies, the abovementioned molecules may actually be metabolized by the liver cytochrome P450 systems and thus their properties may change, thus further research in *in vivo* models to understand the effect of liver metabolism will be required. Moreover, regarding nutritional *in vitro* studies, the bioactive compounds here cited may not all cross the blood brain barrier and thus may not reach the identified targets. Also, dietary bioactive compounds may be transformed by the gut microbiome, as is the case for genistein which is converted into equol, its most active derivative (Matthies et al., 2009). This means further research needs to be performed in order to understand whether food bioactive compounds used in *in vitro* studies are actually the same identical molecules crossing the blood brain barrier after being absorbed in the gastrointestinal tract. Also, most nutritional based studies have focused on the administration of a single bioactive compound. Bioactive compound mixtures, as present in foods, could modify/enhance their activity mutually. For example, as stated in the previous sections, brown seaweeds contain both the carotenoid fucoxanthin and the phytosterol fucosterol and thus may exert a synergistic effect on AD when brown seaweeds are consumed with the diet. However, one caveat of the above described nutritional approaches is that single compounds may have been used at concentrations much higher than those present in foods and thus the observed effects may not be attainable through food intake. In fact, in the previously described *in* vivo lycopene AD study (Sachdeva and Chopra, 2015), lycopene was used at concentrations reaching 4 mg/kg of body weight. In order to reach such dietary intake in humans, more than 2 kg of fresh tomatoes would need to be consumed (Kamiloglu et al., 2014). However, dietary bioactive compound supplementation may overcome this problem. Also, regarding nutritional approaches, intestinal absorption should not be underestimated as is the case for some carotenoids, which are partially absorbed at the intestinal level, as is the case for lycopene, which shows about 20% absorption levels (Moran et al., 2015) and thus, circulating blood carotenoid levels may be too low compared to those used in the previously described studies. Moreover, carotenoid polarity influences greatly their absorption, though dietary fat intake and food matrix also influence carotenoid intestinal absorption, as shown in an* in vitro* Caco-2 intestinal barrier model (Mashurabad et al., 2017). However, carotenoid absorption problems may also be overcome by avoiding oral carotenoid administration, for example, through intraperitoneal administration, as previously stated for crocin *in vivo* studies (Asadi et al., 2015). On the other hand, AD animal studies pose several limitations when compared to human studies, being species-specific differences the most important ones. While extremely useful, currently available transgenic AD models are based on the overexpression of one or more proteins, as suggested from familial AD genetic causes, involved in AD pathology in order for animals to develop AD. However, protein overexpression at much higher than physiological levels, such as APP transgenic mice which develop cerebral amyloidosis (Jacobsen et al., 2014; Neumann et al., 2015) may lead to negative results after compound administration perhaps not necessarily due to compound inefficacy but rather to forced protein expression levels much higher than in AD patients. At such high transgene expression levels, the studied molecules may not be able to exert detectable beneficial effects, while they could do so at physiological protein expression levels. Another issue regards compound metabolism since pharmacological and food bioactive compounds may be differently metabolized by rodents, the most widely used animal model for AD research. Moreover, gut microbiome differences present in humans which can influence bioactive compound metabolism, may not be as sharp in rodents and rodent intestinal permeability may differ from human intestinal permeability for the above mentioned pharmacological and food bioactive compounds. Noteworthy, the amount of pharmacological or food bioactive compounds used in animal studies, when extrapolated to human studies may be quite high. This suggests further consideration is needed when designing human studies. Despite these limitations, animal models continue to be an invaluable resource in AD research. Furthermore, regarding pharmacological approaches in human studies, compound delivery conditions may not be optimal: orally administered compounds may need to be increasingly stable and/or rendered more permeable to brain parenchyma in order to reach their targets in affected areas. Accordingly, pharmacological compound delivery methodologies should be further developed. One last consideration regarding human studies is that gut microbiome differences between different world populations may account for different outcomes in clinical trials when dealing with food bioactive compounds, in case these are metabolized by gut microbiota. Such is the case for genistein conversion into equol, its most active derivative, given that only about 30%–50% of the world population contains gut bacteria able to perform this specific chemical reaction (Atkinson et al., 2005).

## Conclusion

**Figure 2:**
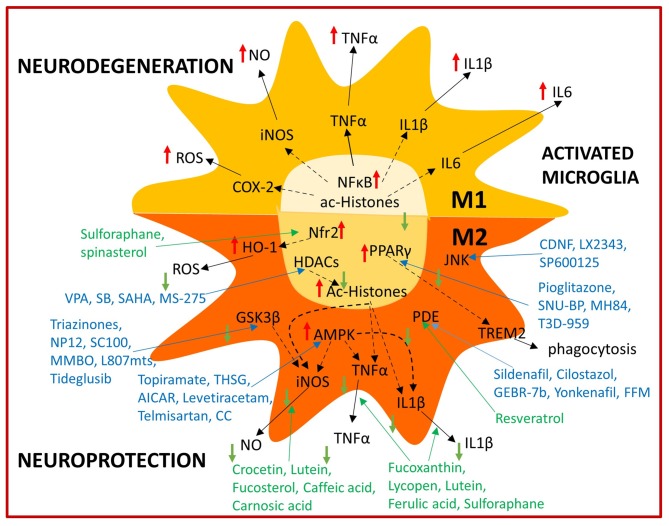
Summary of immunomodulatory bioactive and synthetic compounds. Bioactive (green) and synthetic (blue) compounds are able to modulate microglial activation by shifting from the M1 neurotoxic to the M2 neuroprotective phenotype acting on several intracellular targets.

Pharmacological and nutritional strategies aimed at modulating microglial activation (Figure 2) offer much potential for future brain aging and AD therapy. However, they also represent a big challenge and further research is needed to understand their potential application in humans. Clinical trials focused on immunomodulation targets performed so far, have not resulted in AD patient improvement, however it has to be considered that immunomodulatory strategies may have preventive rather than protective effects. This could derive from the fact that initial AD pathology hallmarks such as Aβ accumulation may start years before symptoms manifest. In fact, protein tangles may already be present in early life and even in healthy aged people (Braak and Del Tredici, 2011), thus perhaps only when a combination of biochemical (Aβ deposition) and cellular factors (Aβ clearance dysfunction) are present, may AD pathology progress. A considerable amount of Aβ turnover is handled by the glymphatic system in the brain and perivascular circulation and dysfunction in these systems may contribute to Aβ accumulation as well (Tarasoff-Conway et al., 2015). Microglial activation thus may be initially beneficial in AD pathology as show by positron emission tomography imaging studies in AD patients and only after becoming chronic it may exacerbate disease (Hamelin et al., 2016; Fan et al., 2017). For example, Aβ direct interaction with the receptor for advance glycation end products (RAGE) increases oxidative stress in neurons, however it also enhances inflammatory response in microglia (Deane et al., 2012). By the time symptoms manifest, microglial dysfunction has already been established and it may be too late to apply current immunomodulation strategies for disease management. However, if compound administration starts as soon as the first biochemical alterations are detected, this may prove an effective preventive strategy. Therefore, a different design of clinical trials directed not towards AD patients, but rather people with MCI should be considered. This approach would hopefully allow to evaluate efficacy from a reduction of MCI individuals undergoing AD and/or a significant delay of this transition. Recent technological advances in MRI allow to discriminate healthy individuals with higher and lower brain levels of Aβ accumulation (Yasuno et al., 2016) which could aid identifying patients with a higher risk of developing MCI and eventually AD in order to better design early intervention clinical trials. If high brain Aβ levels are one of the required factors to trigger AD, yet not the only cause, detecting abnormal Aβ and perhaps tau conformational changes, spreading, deposition and mislocalization at early AD stages may lead to define a time frame in which initial beneficial microglial inflammatory responses are ongoing and immunomodulation strategies thus may prove more effective. Pharmacological therapies are currently experiencing a renaissance, thanks to multitarget drug design, in which already approved or discontinued drugs are fused with other drugs or food bioactive compounds. Thus the obtained hybrid molecule exerts multitarget directed activities derived from the original forming molecules, which may also act on different brain cell populations (Jeřábek et al., 2017). Regarding nutritional approaches, a first step could be adopting, the earlier the better, a healthy eating lifestyle, as close as possible to the Mediterranean diet, in order to increase the intake of bioactive compounds and contribute to healthy aging. Considering the multifactorial nature of cognitive impairment in aging, especially in AD, it is evident that a multitarget-based approach is essential to address this complex condition (Bolognesi, 2013). Interestingly, this multitarget approach can be obtained through either pharmacological or nutritional strategies, however the combined use of both approaches in order to obtain a synergistic effect could be even more effective most probably as a preventive AD/cognitive aging strategy and perhaps also as a therapeutic approach.

## Author Contributions

EP-A contributed to literature search, manuscript writing, editing, revision and final approval. SP contributed to literature search and manuscript writing. FM contributed to literature search and manuscript writing. MV contributed to manuscript editing and revision. MLB contributed to manuscript editing and revision. BM contributed to manuscript editing, revision and figure preparation.

## Conflict of Interest Statement

The authors declare that the research was conducted in the absence of any commercial or financial relationships that could be construed as a potential conflict of interest.
